# Rapid progression of cataract to mature stage after intravitreal dexamethasone implant injection: a case report

**DOI:** 10.1186/s12886-018-1008-7

**Published:** 2019-01-03

**Authors:** Jae Hyung Lee, Jae Yong Park, Jae Suk Kim, Je Hyung Hwang

**Affiliations:** 1 Department of Ophthalmology and Visual Science, Seoul St. Mary’s Hospital, Catholic Institute for Visual Science, The Catholic University of Korea, College of Medicine, Seoul, South Korea; 2 Department of Ophthalmology, Sanggye Paik Hospital, Inje University College of Medicine, 1342 Dongil-ro, Nowon-Gu, Seoul, 139-707 South Korea

**Keywords:** Intravitreal dexamethasone implant, Mature cataract, Ozurdex, Retinal vein occlusion, Macular edema

## Abstract

**Background:**

This study aims to report a case of rapid progression of cataract to mature stage after intravitreal dexamethasone implantation for macular edema due to branch retinal vein occlusion.

**Case presentation:**

A 59-year-old Korean male with complaints of sudden metamorphopsia and reduced visual acuity for three days in the left eye was referred to our clinic. Ophthalmological investigations included fundus photography, fluorescein angiography, and optical coherence tomography. In the left eye, branch retinal vein occlusion with macular edema was observed. We performed intravitreal dexamethasone implantation in the left eye three times within a period of one year. One week after the third intravitreal dexamethasone implantation, grade 1 posterior subcapsular opacity and raised intraocular pressure were observed in the left eye. Three weeks later, mature cataract was observed in the left eye. We performed cataract surgery along with intravitreal ranibizumab injection in the left eye. The procedure was uneventful, and the visual acuity improved postoperatively.

**Conclusions:**

Posterior subcapsular cataract developed due to intravitreal dexamethasone implantation can progress rapidly to mature stage. Therefore, short-term follow-up examinations may be necessary for early diagnosis and treatment of this complication.

## Background

Branch retinal vein occlusion (BRVO) is the second most common retinal vascular disorder and a cause of visual loss due to macular edema (ME) and retinal ischemia. [1, 2] The pathogenesis of ME in BRVO is not fully understood; however, it may result from various causes such as an increase in cytokines and vascular endothelial growth factor (VEGF), [3] high hydrostatic pressure due to increased venous pressure, and dysregulation of endothelial tight junction proteins. [4]

Corticosteroids suppress inflammation and have antiangiogenic properties including the inhibition of VEGF and cytokines involved in the mediation of ME. [5] Facilitating such mechanism, Intravitreal injections of corticosteroid have been shown to be effective in eyes with various diseases, such as retinal vein occlusion (RVO) [6], noninfectious uveitis with posterior segment inflammation, as well as diabetic macular edema. However, such disease generally requires multiple intravitreal injections, and inevitable several adverse effects due to the multiple injections have been noted such as elevated intraocular pressure (IOP) and cataract formation. [6–8]

Sustained-release corticosteroids therefore have been developed to reduce the need for frequent intraocular injections. Dexamethasone intravitreal implant (Ozurdex, Allergan, Inc., Irvine, CA, USA) consists of micronized dexamethasone in a biodegradable copolymer of polylactic-co-glycolic acid, which slowly releases steroids into the vitreous over a period of about six months. [9, 10] It also is less lipophilic and is known to decrease IOP and prevents cataract formation by decreasing accumulation in the trabecular meshwork and the crystalline lens. [11]

Intravitreal Ozurdex implantation is becoming an effective and convenient method for controlling various ocular diseases. However, many complications other than general side effects of the intravitreal corticosteroid have been reported, such as scleral injections, multiple vitreous opacities, retinal aneurysms, as well as keratitis and ptosis. Therefore, the adverse effects of the injection should be considered carefully, and the need for the report of the injection is thoroughly increasing among the ophthalmologists.

The authors have experienced an unusual complication after the intravitreal Ozurdex injection and desire to report the case. In this study, we report a case of rapidly progressed cataract after intravitreal dexamethasone implant injection in a patient with ME with BRVO.

## Case presentation

A 59-year-old Korean male with complaints of sudden metamorphopsia and reduced visual acuity for three days in the left eye was referred to our clinic. His past ophthalmological and other medical history was unremarkable except for hypertension.

**Fig. 1:**
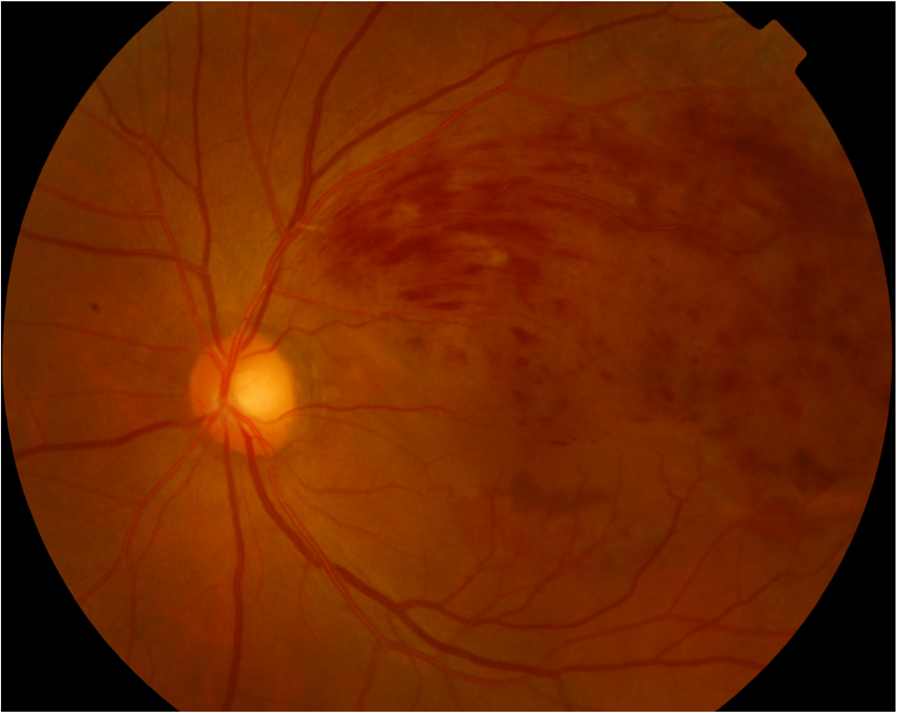
Fundus photograph of a 59-year-old man with branch retinal vein occlusion in the left eye

**Fig. 2:**
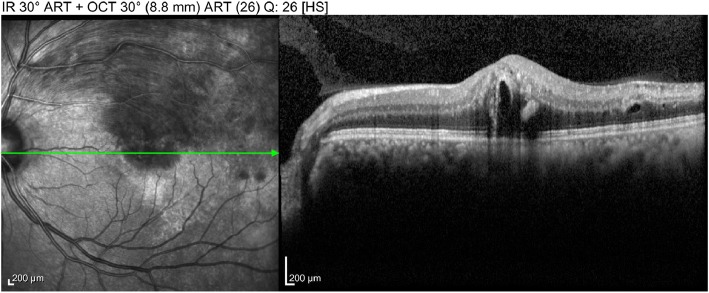
Optical coherence tomography image of a 59-year-old man with macular edema and branch retinal vein occlusion in the left eye

**Fig. 3:**
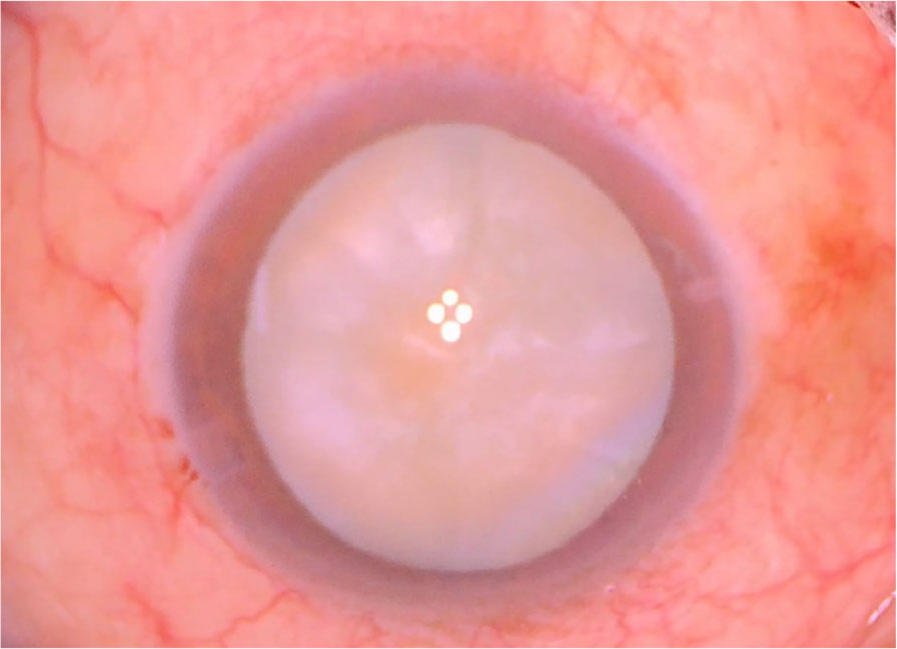
Anterior segment photograph of the left eye at one month after the third intravitreal dexamethasone implantation, showing mature cataract

On examination, the best-corrected distance visual acuity (BCVA) was 20/20 in the right eye and 20/200 in the left eye. On slit-lamp examination, the cornea and conjunctiva were unremarkable, and there was no evidence of active inflammation in the anterior chamber or neovascularization in the iris. Fundus photography and fluorescein angiography showed BRVO in the left eye (Fig. 1). Optical coherence tomography showed ME in the left eye (Fig. 2). We performed intravitreal dexamethasone implantation and scatter laser photocoagulation in the left eye. The intravitreal dexamethasone implant injection was performed inferotemporally, 3.5 mm from the limbus. The implant was properly positioned in the vitreous chamber after the injection. One month after the intravitreal dexamethasone implantation, a decrease in the ME and an improvement of the BCVA to 20/40 was observed on left eye examination. Three months after the intravitreal dexamethasone implantation, recurrence of the ME and deterioration of the BCVA to 20/200 was observed on left eye examination. Therefore, we performed the second intravitreal dexamethasone implantation in the left eye in the same manner. One month after the second intravitreal dexamethasone implantation, the ME improved and the BCVA was 20/60 in the left eye. The ME recurred and the BCVA was 20/200 about four months after the second intravitreal injection. Therefore, we performed the third intravitreal dexamethasone implantation in the left eye in the same manner. Every intravitreal injections and the consequent follow-up examinations were performed by an experienced, single vitreoretinal specialist. On every follow-up examinations performed a day after the three dexamethasone implantations, the implant was positioned properly in the vitreous chamber, away from the crystalline lens. On slit-lamp examination performed one week after the third injection, grade 1 posterior subcapsular opacity was observed and the IOP was 42 mmHg by Goldmann applanation tonometer; however, there was improvement in the ME and the BCVA was 20/100. He was treated with oral acetazolamide, topical dorzolamide/timolol, and topical bimatoprost in the left eye. His IOP decreased to 18 mmHg in the left eye. He was discharged and prescribed topical dorzolamide/timolol and topical bimatoprost in the left eye and oral acetazolamide 10 mg/kg three times a day. Three weeks after the treatment, on slit-lamp examination, we observed that the posterior subcapsular cataract had progressed to mature stage (Fig. 3); anterior chamber was shallower than that observed in the previous examination. The IOP was 18 mmHg and the BCVA was reduced to hand motion in the left eye. Phacoemulsification and the consequent posterior chamber intraocular lens implantation was performed to treat the mature cataract, and intravitreal ranibizumab was performed in order to decrease the remnant ME in the left eye. The procedure was uneventful. His BCVA in the left eye was 20/60 one week after the procedure.

## Discussion and conclusions

Significant improvements in visual acuity have been observed with intravitreal dexamethasone implantation in eyes with visual impairment due to ME associated with RVO. [5] However, cataract development is a well-documented consequence of the intravitreal steroid injection. [12, 13] The incidence of cataract progression after intravitreal dexamethasone implantation is 2.0 to 58.8%. [14–17] Prolonged and repeated exposure to intravitreal dexamethasone implant is associated with the development or progression of cataract. [18] Haller et al. reported a positive correlation between the intravitreal dexamethasone implant and cataract progression. [19] Reid et al. reported that an increased incidence of cataract surgery was observed with the second and third implant groups compared to the first implant group. [20] In our case, the first and second implants were not associated with the development or progression of cataract; however, the third implant resulted in cataract, which rapidly progressed to mature stage. Moisseiev et al. reported that the mean time between the dexamethasone implant and cataract surgery was 28.3 months and only one patient underwent cataract surgery within one year of the dexamethasone implantation [14]. Reid et al. reported that the average time to cataract surgery after the first dexamethasone implant was 377 days. [20] The time lapse between the dexamethasone implant and cataract surgery was 315 days in our patient. The relationship between the number of injections as well as the time lapse after the injections and the formation of cataract could not precisely be verified at present. Other previous studies have described cataract formation after the intravitreal dexamethasone implantation; [14–20] however, there have been no reports of rapid progression of cataract to mature stage within three weeks of intravitreal dexamethasone implantation.

Other causes of the cataract progression may be possible. One possible cause of the cataract progression due to the traumatic rupture of the lens capsule resulting from the dexamethasone implantation. It is possible that a resultant small rupture of the capsule may have promoted a formation of traumatic cataract. This possibility, however, is relatively low because any lens capsule defect was not visible during the surgery. Also, the close distance between the crystalline lens and the dexamethasone implant should be considered. Although the follow-up examinations after the injection revealed enough distance secured from the crystalline lens and the implant, the implant may have wondered around the vitreous cavity to occasionally narrow the distance.

In conclusion, to the best of our knowledge, this is the first reported case of rapid progression of cataract to mature stage after intravitreal dexamethasone implantation. Usually, cataract formation after intravitreal dexamethasone implantation is a slow process. Therefore, cataract surgery is usually required after a long follow-up period. However, short-term follow-up examinations are required after the formation of cataract following intravitreal dexamethasone implantation as there may be a rapid progression of the cataract as observed in the present case. Such cases may require urgent surgery depending on the stage of the cataract.

## Abbreviations

BCVA: best-corrected distance visual acuity
BRVO: branch retinal vein occlusion
IOP: intraocular pressure
ME: macular edema
RVO: retinal vein occlusion
VEGF: vascular endothelial growth factor
